# Practice-based learning: an appropriate means to acquire the attitude and skills for evidence-based medicine

**DOI:** 10.5116/ijme.5ee0.ab48

**Published:** 2020-07-24

**Authors:** Tamara E.T. van Woezik, Jurriaan P. Oosterman, Rob P.B. Reuzel, Gert-Jan van der Wilt, Jur J. Koksma

**Affiliations:** 1 Radboud University Medical Centre, Nijmegen, the Netherlands

**Keywords:** Practice-based Learning, evidence-based medicine, critically reflective practitioner, critical thinking

## Abstract

**Objectives:**

To evaluate a practice-based, self-directed EBM-course in an undergraduate medical curriculum in terms of EBM attitude and motivation beliefs.

**Methods:**

This study was conducted in a 4-week course of the first-year undergraduate medical curriculum, which takes place twice in an academic year. One group of students (n=210) received a normal EBM-module in November. A practice-based EBM-module was implemented in January for another group of students (n=130). We approached all students following the courses for participation in our research project. In a quasi-experimental design, a validated survey was used to assess students' EBM task value and self-efficacy on a 7-point Likert-scale. In the experimental group, complementary qualitative data were gathered on attitude and motivation by open evaluative questions.

**Results:**

Overall response rate was 93,5%, resulting in 191 students in the control group and 127 students in the experimental group. We did not find differences between the groups in terms of EBM task value and self-efficacy. However, the experimental group showed a higher increased perception of the importance of EBM in decision making in clinical practice (60.0% vs 77.2%; χ^2^(1, N=318) = 8.432, p=0.004). These students obtained a better understanding of the complexities and time-consuming nature of EBM in medical practice.

**Conclusions:**

The practice-based EBM-course helps students to reflect on practice and knowledge critically. Our findings indicate that integrating clinical practice in the undergraduate learning environment fosters attitude and motivation, suggesting that practice-based learning in EBM education may advance student development as a critically reflective practitioner.

## Introduction

Over the last decades, Evidence-based medicine (EBM) has become part of medical curriculum attainment targets across the globe. Although EBM started as a promising movement, today it faces accusations that much EBM research would be irrelevant, unreflective or sloppy.^1^^-^^4^ The very approach to EBM would be reductionistic, insufficiently acknowledging the rich and complex medical context which it is meant to support. Nevertheless, there is an ongoing need for useful scientific evidence that practice and patients benefit from. EBM is therefore still relevant to medical practice, and EBM knowledge and skills are important for future professionals. However, simply following guidelines based on EBM is not the way forward.^5^ Instead, EBM should aid in advancing medical practice as a learning environment open to various ways of knowing and contrasting perspectives.^6^

Thus, in the light of these developments, it is imperative for EBM instructors to use EBM in such a way that it stimulates a critical, reflective mindset. It has already been shown that obtaining EBM knowledge and skills alone is not enough to ensure proper use of EBM in practice.^7^ Early EBM education has different effects on learners' attitudes such as self-efficacy beliefs,^7^^, ^^8^^, ^^9^ revealing a potential to foster a critically reflective attitude. Nevertheless, we do not know what educational strategies help undergraduate students to acquire not only skills and knowledge but also the necessary attitude and motivation to make use of EBM in medical practice properly.^10^^, ^^11^

We do know that early exposure to real clinical settings stimulates professional identity development.^12^ A systematic review investigating effective teaching strategies points out that practice-based education strategies promote a reflective attitude.^13^ Practice-based education involves learning from experience 'in the real world', which means that students spend time in genuine professional environments.^14^ Here, they encounter problems or questions, which they take back to the classroom to work on.^15^ Their results are then taken back to practice, which means both education and clinical practice may actually benefit. The idea is that this type of education will help students think more critically and be more motivated.^16^ Furthermore, studies suggest that clinical experience involved in practice-based learning helps promote EBM competency.^17^^-^^19^ This suggests that a combination of practice-based learning and EBM education could help to promote good use of EBM in medical practice. Therefore, we designed a practice-based, EBM-course for first-year medical students. The research objective of this study is to evaluate the effects of this course on students' EBM attitude and motivational beliefs.

## Methods

### Study design

A quasi-experimental design was used to evaluate the effects of a practice-based EBM-course on students' EBM attitude. Pre-test and post-test scores were obtained using a survey on EBM task value and self-efficacy with additional questions on EBM attitude and beliefs. We included first-year medical students to investigate the effect of a practice-based EBM-course (experimental group) or non-practice-based EBM-course (control group) on the students' EBM attitude.

### Participants

First-year medical students of the Radboudumc participated in EBM education. The course, named 'Doctor in context', featured the basics of Epidemiology, Medical Statistics and EBM. The curriculum was divided into cohort groups that follow courses in different sequences. Students can follow the course 'Doctor in context' in two different periods. This made it possible to study two naturally occurring groups: one cohort as a control group (n=130) with the regular course in November, and the other cohort as the experimental group (n=210) with a practice-based EBM-course in January. The control group received an alternative non-practice-based EBM assignment, comparable to the content of the practice-based EBM-course. The Netherlands Association for Medical Education (NVMO) ethical review board and the University management team approved this study (NVMO-ERB number 200). Both groups were informed and provided written informed consent before participation.

### Intervention

The six-year Doctor of Medicine program of the Radboudumc comprises 3 years of undergraduate education and 3 subsequent years of internships, during which students encounter different medical disciplines. The year 1 practice-based EBM-course consisted of three contact moments between a student group (n=15) and a clinical tutor, over three weeks at a clinical unit, see Table 1. The practice-based EBM-course was supervised by two of the same teachers as the control group and clinicians.

In the experimental group, students (in subgroups of five) witness a clinical case discussion during a handover meeting in the clinic and formulate clinical questions about therapy, diagnosis, prognosis or etiology from one of the more complex patient cases. Students had to answer these clinical questions according to the method of evidence-based medicine: step 1) Formulating a foreground question; step 2) Searching for scientific literature (PubMed, e.g.); step 3) Critical appraisal of scientific literature; step 4) Presenting and formulating the answer. Both the students and the clinical instructors who participated in the course received individual written instructions.

### Control group

The control group followed a program similar to the experimental group regarding the experience with evidence-based medicine. However, this program was not practice-based, so the control group did not have any meetings at clinical units, but had meetings in the classroom only (see Table 1). The teachers in this regular course had a background in methodology and research.

### Instruments

To measure students' attitude towards EBM we used the validated survey of Spek and colleagues.^7^^,^^8^ It consists of 20 items, with a 7-point Likert Scale (1 = strongly disagree, 4 = neutral, 7 = strongly agree). The items are divided over two components representing students' EBM task value and self-efficacy, with 11 and 9 items respectively. For EBM task value, positive items are included, for instance: "I believe it is important to encourage other students to search for scientific studies" and reversed items are included such as: "I believe EBM is too time-consuming and does not outweigh the benefits". For EBM self-efficacy all items are reversed, for instance: "I believe my abilities to find scientific evidence are not adequate". Small adjustments were made in the survey to make it suit the local context. We added some close-ended questions about the personal background (sex, age, education) as well as two questions where students could rate EBM as a method and add written comments.^20^

### Data collection

Data collection did not differ between the control group and the experimental group. The information letter and informed consent were provided online, two days in advance, to allow for reading and decision time. The information letter and informed consent were also available in print at the start of the course, and one of the researchers (JO) was present to answer any questions. After filling out the informed consent, students received the survey on EBM task value and self-efficacy. Students received a paper version of the EBM survey, both at the start of the course and afterwards, during class meetings with a tutor present. Supervision was done by a tutor to avoid consulting. Answers to the questionnaire were then entered into IBM SPSS version 22, with random codes to pseudonymize the collected data but allow for pre- and post-test comparisons.

**Table 1:** Outline of the practice-based and regular EBM-course

Step	Experimental group: practice-based EBM- course	Step	Control group: non-practice-based, regular EBM- course
Step 1	Formulating a foreground question	Step 1	Formulating a foreground question
Contact moment 1	1a. Transfer meeting at a clinical unit; 1b. Selection of patient cases with clinical questions in collaboration with a clinician; 1c. Formulation of foreground question and search strategy;	Class meeting 1	1a. Opening lecture; 1b. Selection of case from theoretical examples; 1c. Formulation of foreground question and search strategy;
Step 2	Searching in the literature	Step 2	Searching in the literature
	2a. Searching in different databases (e.g. Google, PubMed, UpToDate, Cochrane); 2b. Selection of articles & critical appraisal;		2a. Searching in different databases (e.g. Google, PubMed, UpToDate, Cochrane); 2b. Selection of articles & critical appraisal;
Step 3	Critical appraisal of the literature	Step 3	Critical appraisal of the literature
Contact moment 2	3a. Feedback and discussion on the first results with a clinician; 3b. Adjustments of results and/or selection of articles;	Class meeting 2	3a. Feedback and discussion on the first results with a teacher; 3b. Adjustments of results and/or selection of articles;
Step 4	Presentation and formulating an answer	Step 4	Presentation and formulating an answer
Contact moment 3	4a. Report and presentation of the results followed by discussion, feedback and reflection with clinical unit and clinician.	Class meeting 3	4a. Report and presentation of the results followed by discussion, feedback and reflection in the classroom.

### Analysis

Quantitative data were analyzed using SPSS statistics version 20.0. We calculated Cronbach's α for the task value and self-efficacy scales. After inversion of items with a negative relation to the component, differences in the mean scores for task value and self-efficacy between both groups were tested using the independent t-test. Pre-post-test analysis within groups was performed using a paired sample t-test. An independent samples t-test was used for comparison between the groups. Students' post-test explanations were open-ended questions. The answers were coded based on the themes they represented. These themes were then categorized, rated and analyzed by chi-square- and point-biserial correlation (rpb).

## Results

Table 2 shows the pre- and post-test results of both groups. The control group consisted of a total of 127 students and the experimental group of 191 students. The response rate (˃ 90%) was good in both groups. Both components task value and self-efficacy had good reliabilities: Cronbach’s α = 0.78 (95% CI 0.76-0.81) and 0.81 (95% CI 0.79-0.83) respectively. Histograms and boxplots of all items showed a normal distribution and no statistically significant outliers. Student's characteristics did not differ between groups and were in line with the overall curriculum with a mean age of 18.7 years and 65% women, 35% men.

**Table 2:** Mean scores "Task value" and "Self-efficacy" (7-point Likert scale, 1 -7)

Groups	N	Pretest mean (SD)	Posttest mean (SD)	Paired t-test t-score	*p-*value
Task value					
Control Group	127	4.58 (0.61)	4.57 (0.60)	*t*_(__126)_= 0.355	*p=*0.723
Experimental Group	191	4.57 (0.57)	4.52 (0.63)	*t*_(__190)_=1.005	*p=*0.316
Self-efficacy					
Control Group	127	3.49 (0.96)	3.91 (0.89)	*t*_(126)=_-7.085	*p*<0.001
Experimental Group	191	3.55 (0.83)	3.93 (0.76)	*t*_ (190)=_-7.638	*p*<0.001

The mean pre-test and post-test scores on both components task value and self-efficacy showed no significant differences between both groups. After the EBM-course, the mean post-test scores on task value were not significantly higher for the control nor the experimental group (respectively 4.58 vs. 4.57; *t*_(126)_= 0.355, p=0.723, and 4.57 vs. 4.52; *t*_(190)_=1.005, p=0.316). Scores on self-efficacy were significantly higher in both groups (3.49 vs. 3.91; t_(__126)_=-7.085, p< 0.001, and 3.55 vs. 3.93;* t*_(190)_=-7.638, p< 0.001), see Table 2.

After the regular course and the EBM-course, students in both groups were more positive regarding how they feel about EBM (control group: 3.96 vs. 4.53; *t*_(122)_=-6.067, p<0.001, experimental group: 3.86 vs. 4.44; *t*_(185)_=-6.272, p< 0.001) and how they perceive it as a method (control group: 6.83 vs. 7.23; *t*_(117)_= -4.267, p< 0.001, experimental group: 6.83 vs. 7.13; *t*_(179)_= -4.400, p<0.001), see Table 3. Mean scores were similar between both groups.

**Table 3:** Students' ratings regarding EBM

Groups	N	Pretest mean (SD)	Posttest mean (SD)	Paired t-test t-score	*p-*value
How do you feel about EBM? (1-7)					
Control Group	123	3.96 (1.02)	4.53 (0.75)	*t*_ (122)=_ -6.067	*p*<0.001
Experimental Group	186	3.86 (1.14)	4.44 (0.89)	t_ (185)=_ -6.272	*p*<0.001
How do you regard EBM as a method? (1-10)					
Control Group	118	6.83 (0.92)	7.23 (0.83)	t_ (117)=_ -4.267	*p*<0.001
Experimental Group	180	6.83 (0.87)	7.13 (0.82)	t_ (179)=_ -4.400	*p*<0.001

Table 4 shows the explanations the students gave about their ratings on EBM. Students were also more likely to have an increased perception of the importance of EBM in medical education and objective decision making in clinical practice (χ^2^(1, N=318)=8.432, p=0.004). At the same time, students in the experimental group were more likely to doubt the efficacy of EBM in practice (χ^2^(1, N=318)=9.747, p=0.002). Besides this, students in the experimental group learned that EBM could be time-consuming (χ^2^(1, N=318)=11.670, p=0.001).

**Table 4:** Students' post-test explanations regarding EBM as a method

How do you regard EBM as a method?	Control group (n=127, %)	Experimental group (n=191, %)	Pearson-Chi square, *p-*value
R1. Patient perspective also important	12.3	10.2	c^2^_(1, N=__318)_=0.547, *p=*0.470
R2. EBM method is difficult	16.2	9.6	c^2^_(1, N=318)=_3.011, *p=*0.087
R3. EBM important in medical education / clinical practice	60.0	77.2	c^2^_(1, N=318)=_8.432,* p=*0.004
R4. EBM is time-consuming	8.5	23.4	c^2^_(1, N=318)=_11.670, *p=*0.001
R5. Not sure about efficacy of EBM in practice	14.6	27.9	c^2^_(1, N=318)=_9.747, *p**=***0.002

Point-biserial correlation (rpb) analysis of students explanations and the components task value and self-efficacy showed significant but weak associations in both groups. The experimental group was more likely to have an increased perception of the importance of EBM in medical education and objective decision making in clinical practice. This is also illustrated by item 10 of the component task value, which showed a relatively significant difference between the post-test mean scores of both groups. More students from the experimental group (mean = 4.14, SD = 1.12) agreed with the statement "I believe EBM is too time-consuming and does not outweigh the benefits" than the control group (mean = 3.72, SD = 1.18).

## Discussion

In this study, we investigated the effect of a practice-based course on students' attitude and behavior regarding EBM. We hypothesized that the integration of EBM with clinical practice helps students to develop increased task value and self-efficacy for EBM, and develop a more positive attitude towards EBM. EBM self-efficacy did increase, whereas EBM task value did not. EBM attitude results suggest that practice-based learning leads to an increased understanding of the complexities of using EBM in medical practice.

### Attitudes about EBM

Students in the experimental group showed an increased perception of the importance of EBM in medical education and informed decision making in clinical practice. Some of them were also more critical about the effectiveness and feasibility of EBM in practice. They experienced that it can be time-consuming to find high qualitative scientific literature in the right domain, that critical appraisal of articles can be difficult, and that translation of the results to their patient case is far from straightforward. At the same time, they also acknowledged that more practice could be beneficial.

Students were studying for merely three to five months, and this course was their first encounter with medical science and statistics, which is generally associated with particular anxieties^21^ and higher cognitive load.^22^ We think this makes the results of the experimental group all the more interesting, for it suggests that the practice base of their education makes them look beyond their own immediate demands.

When we asked the students about the feasibility of EBM as an effective method in clinical practice, they expressed doubts. When they could not find relevant and satisfactory results for their patient case, students tended to become less motivated. For students to stay motivated to work on relatively complex tasks, it is incumbent upon teachers to help them build or maintain a sense of relatedness and competence.^23^^, ^^24^ Indeed, our findings highlight the importance of creating the right learning environment, if one wants to maintain a balance between student levels of autonomy and feeling competent. This is in line with findings from one of our earlier studies on innovative education methodology.^25^

As professional role models and teachers at the same time, doctors can make a difference here. When they involve students and show them how they struggle with the scientific uncertainties of complex medical practice, this creates a shared and meaningful experience. In turn, this helps students to mitigate their anxieties and engage in critical thinking, which is best promoted in these specific contexts.^26^ Furthermore, by regularly exposing students to practice-based EBM formats during their study, they will not only learn how to find more relevant articles and answers but also experience how to make meaningful use of EBM methods in the wider context of patient care. More research should answer the question whether this will continue to motivate them throughout their professional career.^27^^-^^29^ At least, this study's results indicate that practice-based learning rightfrom the start of medical school may set the narrative in a motion of becoming such a critically reflective practitioner.^30^^-^^32^ 

### EBM task value and self-efficacy

EBM self-efficacy and task value are believed to predict the further use of EBM in practice, and as such are important outcome measures of an EBM course. Self-efficacy tells something about how competent a student feels with respect to completing a task and is linked to the perception of her or his ability to perform a certain future task.^33^ In both the experimental group and the control group, mean pre-test and post-test scores differed significantly on the component EBM self-efficacy. This may indicate that the course led to increased feelings of competence, regardless of the educational method.

Minor changes were measured on the other component, EBM task value. Task value refers to the perceived meaning a leaner ascribes to a task. In the study of Spek et al., no changes were measured on both components between different years of students either.^8^ This raises the question if the component EBM task value is sensitive enough to measure change. However, a more probable explanation is both their and our courses had no effect on EBM task value. Thus, the question remains unanswered how we can increase EBM task value for students. One way forward may be to change toward educational designs that we know increase motivation in general.

### Patient included

In the new curriculum of the Radboudumc, patients are more involved in education. This patient included curriculum might help to foster EBM task value. Students spend more time in clinical practice, they are present during transfer moments, they talk to medical doctors, they meet patients and involve them in their educational assignments. We see that this fosters motivation, but also helps establish the practice base to make learning relevant for the future profession.^34^ Involving not only researchers but also patients in an EBM course might have similar results.

In another study, we have found that students in our institution score quite low on self-reported use of critical thinking strategies, at least in the first two years of their education.^25^ The current study shows that practice-based learning may be valuable in that respect. Including patients will enrich this practice base in education, making it more urgent and intellectually stimulating to think about what evidence can bring to the table of shared decision making with the individual patient. Moreover, it will strengthen the affective component in learning, which can aid the learning process. This may also help students appreciate critical thinking 'in action', and not view it in an overly cognitive, instrumental way.^35^

In terms of education method, this study shows that the integration of basic sciences like EBM with clinical practice from early in the curriculum might be a promising strategy to foster critical reflection. Ten Cate and Scheele advocate practice-based learning in which supervision is progressively reduced, entrusting students over time with more responsibilities.^36^ This approach aligns with self-directed learning, which is also often incorporated in medical education to foster development of critically reflective practitioners.^37^ The current study shows that practice-based learning may increase self-efficacy and critical thinking, suggesting a close relation between practice-based learning and self-directed learning. The underlying mechanism might be that a practice base increases feelings of relatedness and competence by having role models (clinicians) encouraging students. Indeed, the importance of relatedness and competence for self-directed learning has been indicated before.^25^^,^^28^

### Limitations, implications, and future research

We studied two naturally occurring cohort groups, which meant that we had no problems with randomization or inclusion of participants. A disadvantage of this method is that the students in the experimental group did have additional experience, namely two courses over two months. We tried to overcome these differences by administering a pre-test, which indeed shows little difference between the groups.

Although the survey has certain benefits for evaluation, more research on its reliability and validity is needed. A change toward more practice-based learning can help to promote critical reflection. However, research in education often shows that its outcomes are dependent on context. It will be valuable to look at how programs or policy interventions give rise to certain outcomes, in terms of the underlying mechanisms involved.^38^ We think that such scrutiny will lead to a better understanding of practice-based learning, and useful suggestions for course design.

## Conclusions

The aim of this study was to develop and execute a practice-based EBM-course in the undergraduate medical curriculum and to evaluate its effect on students' EBM attitude. The students in the control group and in the practice-based EBM-course showed the same increase in EBM self-efficacy and the same lack of effect on EBM task value. Students in the experimental group did show a heightened awareness of problems regarding the pursuit of EBM in clinical practice. Coping with uncertainties of complex practice should be explicitly addressed, to help students stay motivated and develop critical reflection. This study shows that practice-based courses can help to educate our students as critically reflective practitioners, even at the very beginning of medical school.

### Acknowledgements

We thank Prof. Dr. A. Verbeek and Dr. J. Van Dijck for their contribution to making this study possible in the undergraduate medical curriculum of the Radboudumc Nijmegen. The authors would like to thank all the students for their participation, with special thanks to all clinical tutors: Dr. J. Deinum, Prof. Dr. M. OldeRikkert, Drs. A. Van Linge, Dr. J. Draaisma, Prof. Dr. H. Marres, Prof. Dr. F. Lotgering, Dr. A. Timmer-Bonte, Prof. Dr. J. Keunen, Prof. Dr. J. Drenth, Prof. Dr. B. Van Engelen, Dr. B. Post, Dr. D. Oosterbaan, Prof. Dr. L. Schultze Kool, Dr. L. Peters-Bax, Dr. W. Klein, Dr. T. Jansen, Dr. J. Groothuis. For statistical advice we thank dr. R. Donders.

### Conflict of Interest

The authors declare that they have no conflict of interest.
